# In Vitro/In Vivo Evaluation of Clomipramine Orodispersible Tablets for the Treatment of Depression and Obsessive-Compulsive Disorder

**DOI:** 10.3390/ph16020265

**Published:** 2023-02-09

**Authors:** Shazia Akram Ghumman, Huma Hameed, Sobia Noreen, Sami A. Al-Hussain, Rizwana Kausar, Ali Irfan, Ramla Shabbir, Maria Rana, Amina Amanat, Magdi E. A. Zaki

**Affiliations:** 1 College of Pharmacy, University of Sargodha, Sargodha 40100, Pakistan; 2 Faculty of Pharmaceutical Sciences, University of Central Punjab, Lahore 54000, Pakistan; 3 Institute of Chemistry, University of Sargodha, Sargodha 40100, Pakistan; 4 Department of Chemistry, College of Science, Imam Mohammad Ibn Saud Islamic University (IMSIU), Riyadh 11623, Saudi Arabia; 5 ILM College of Pharmaceutical Sciences, Sargodha 40100, Pakistan; 6 Department of Chemistry, Government College University Faisalabad, Faisalabad 38000, Pakistan; 7 Faculty of Pharmacy, University of Lahore, Lahore 54760, Pakistan; 8 Riphah Institute of Pharmaceutical Sciences, Riphah International University Lahore Campus, Lahore 54000, Pakistan

**Keywords:** clomipramine (CLP), *Plantago ovata* mucilage, orodispersible tablets, disintegration time, in vitro drug release, in vivo antidepressant activity

## Abstract

The first and only antidepressant drug on the market with solid proof of clinically significant serotonin and noradrenaline reuptake inhibition is clomipramine (CLP). However, significant first-pass metabolism reduces its absorption to less than 62%. It is heavily protein-bound and broadly dispersed across the body (9–25 L/kg volume of distribution). The purpose of this research was to formulate CLP orodispersible tablets that immediately enable the drug to enter the bloodstream and bypass systemic portal circulation to improve its bioavailability. A factorial design was employed using varied amounts of *Plantago ovata* mucilage (POM) as a natural superdisintegrant, as well as croscarmellose sodium and crospovidone as synthetic disintegrants. Their physiochemical compatibility was evaluated by FTIR, DSC/TGA, and PXRD analysis. The blend of all formulations was assessed for pre- and post-compaction parameters. The study found that tablets comprising *Plantago ovata* mucilage as a superdisintegrant showed a rapid in vitro disintegration time, i.e., around 8.39 s, and had an excellent dissolution profile. The anti-depressant efficacy was evaluated by an open-field test (OFT) and the forced swimming test (FST) was applied to create hopelessness and despair behavior as a model of depression in animals (Albino rats). The in vivo study revealed that the efficiency of the optimized formulation (F9) in the treatment of depression is more than the marketed available clomfranil tablet, and may be linked to its rapid disintegration and bypassing of systemic portal circulation.

## 1. Introduction

A broad spectrum of pharmaceutical studies has recently been focused on designing innovative dosage compositions; in addition, most such attempts are now being aimed toward patient compliance whenever it concerns living standards [1]. Orodispersible tablets (ODTs) are oral unit solid dose forms that rapidly dissolve in the mouth to facilitate ingesting and prevent choking. Additionally, these dosage forms target both the pediatric and geriatric populations and have exhibited improved patient satisfaction [2,3]. Prenatal, elderly, paraplegic, or individuals having neurodevelopmental disorders might encounter difficulties ingesting traditional pills/caplets and oral fluids, which could result in poor treatment for patients suffering recurrent vomiting, rapid outbreaks of hypersensitivity, and sneezing [4]. ODTs may be appropriate for impotence medications, antipsychotics, and cardiorespiratory treatments, including anti-allergy medications. ODTs are also known as either fast dissolving tablets (FDTs), rapidly dissolving tablets (RDTs), fast disintegrating, rapid melt, immediately disintegrating tablets (IDTs), or amorphous tablets [5]. Saliva quickly enters the non-visible pores, that became visible upon hydration in such ODTs, when they are placed in the oral cavity, leading the tablets to quickly dissolve without the individuals needing to chew anything. The average time for ODTs that dissolve or break down in the mouth before being swallowed is less than three minutes [6]. Orodispersible tablets significantly showed better bioavailability of active ingredients than other kinds of tablets because of their rapid disintegration time [7]. For patients having depression or disabilities, and pediatric, geriatric, and pregnant women with nausea, vomiting, or swallowing issues, orodispersible tablets are a very practical and popular therapeutic strategy [8,9,10]. Additionally, ODTs also showed the benefit of avoiding the first-pass effect and thus increasing the bioavailability of the active ingredient [11].

The release/discharge feature of the active ingredient from a pharmaceutical product is significantly influenced by the rate at which tablets disintegrate. This rate is based on the characteristics of the disintegrant that enhances breakdown [3,12]. Disintegrants are compounds or material blends that help tablets and other pharmaceutical formulations break down and disintegrate into smaller pieces [13]. Disintegrants are thus tasked with ensuring that tablets break down into smaller pieces when they encounter water, resulting in a higher disintegration rate [14]. Disintegrants increase the effectiveness of solid drug formulations by shortening the time required for disintegration, which in turn speeds up dissolution and improves the bioavailability of the active ingredient [15]. Disintegrants that are synthetic or semi-synthetic are frequently utilized for making tablets. Crospovidone, with a high compressibility, low friability, and high breaking force, is one of the primary members of this category, along with sodium starch glycolate and croscarmellose sodium [16]. However, natural disintegrants have more advantages than synthetic ones, since they are locally readily available, eco-friendly, biocompatible, sustainable, and more affordable as compared to synthetic alternatives [17].

In recent times, naturally extracted, isolated, and highly purified plant-based additives have come in demand and replaced synthetic additives because they are non-toxic, low-cost, readily available, and non-irritating. These natural substances have several advantages compared to semi-synthetic and synthetic materials [18,19,20]. *Plantago ovata* powder is primarily comprised of the peel of dried seeds. *Plantago ovata* mucilage is polar, with a lot of highly twisted neutral arabinoxylan and non-reducing terminal carbohydrate residues. The weight of *Plantago ovata* increases up to ten times because of rapid water uptake [17]. *Plantago ovata* mucilage contains 10–30% hydrocolloids as water-soluble polysaccharides that, in contact with water, make flakes of mucilage. This mucilage breaks upon hydration, yielding polysaccharides such as xylose, arabinose, galacturonic acid, rhamnose, and galactose [21]. These substances could be used as natural disintegrants in the production of pharmaceuticals, and are preferable to synthetic ones [22].

To reduce the number of experiments and to find out the best quality of the formulation, the most promising experimental design is necessary [23,24]. The central composite design (CCD) can be performed on precisely selected variables that affect the responses significantly. It mainly represents a set of statistical and mathematical models/methods, that are used for the analysis of the relationships between one or more dependent variables (measured answers) and a number of independent variables in order to obtain an optimal formula. We applied CCD to obtain the optimal formula of the experimental conditions, which minimized or maximized a response of the system and the modifications of the response surface in the domain of independent variables [25,26].

Tricyclic antidepressants, which include clomipramine (CLP), are being used to treat and reduce the signs of depression and obsessive-compulsive disorder. Additionally, by inhibiting the receptors for norepinephrine and serotonin, they prevent their reuptake by the central nervous system [27]. The oral bioavailability of clomipramine is reduced to less than 62% due to significant first-pass metabolism. Clomipramine has a plasma half-life of 20–24 h and follows first-order elimination pharmacokinetics [28]. The goal of this study was to use a direct compression method to improve its bioavailability and optimize CLP ODTs with varying ratios of three distinct SDs: croscarmellose sodium (CS), crospovidone (CP), and *Plantago ovata* mucilage (POM). The formulation quality was evaluated by applying pre- and post-compression tests, such as bulk/tapped density, firmness, friability, in vitro dissolution, and disintegration time. We further used the forced swimming test (FST) as a model of depression, and the open-field test (OFT) as a separate indicator of general activity, with the goal of determining the efficacy of CLP ODTs to cure depression.

## 2. Results and Discussion

In the present work, orodispersible tablets of clomipramine HCl were formulated by applying the direct compression method using two synthetic superdisintegrants (croscarmellose sodium and crospovidone) and one natural superdisintegrant (*Plantago ovata* mucilage).

### 2.1. Physicochemical Characterization of Superdisintegrants

Selected acceptable SDs in clomipramine mouth dissolving tablets were evaluated for different parameters such as percentage loss on drying, bulk density, tapped density, Carr’s index, Hausner’s ratio, and angle of repose. Table 1 represents the estimated values of all the above-mentioned parameters.

### 2.2. Drug–Superdisintegrant Compatibility Study

#### 2.2.1. Fourier Transform Infrared Spectroscopy (FTIR) Analysis

**Figure 1:**
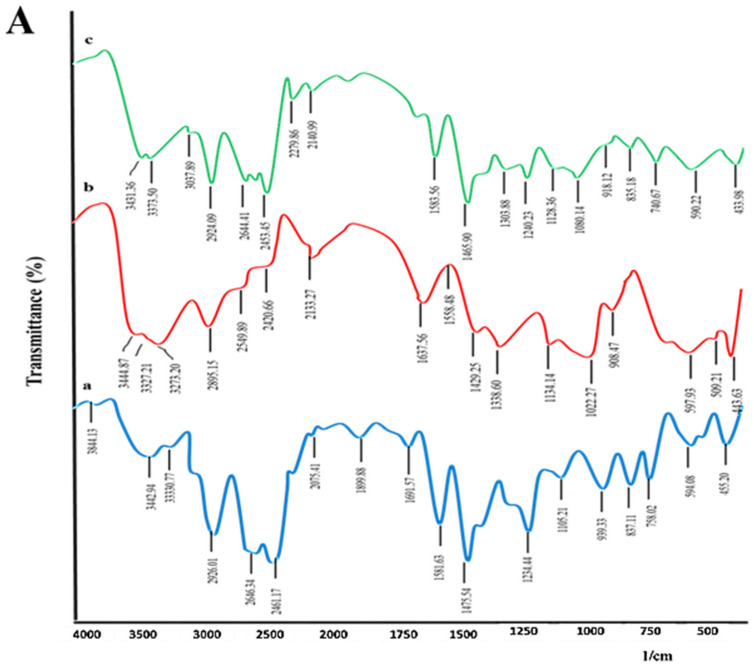

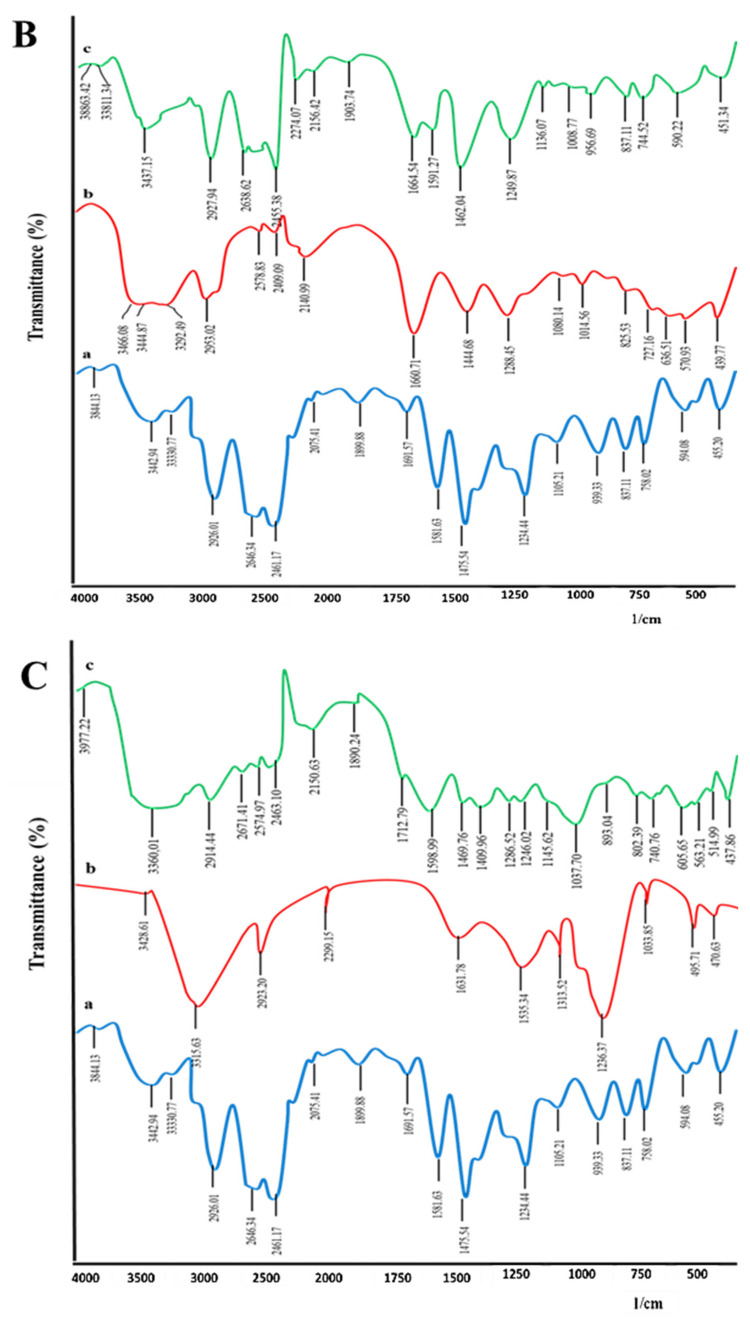
(**A**). FTIR overlay display of (a) clomipramine, (b) croscarmellose sodium, and (c) their mixture; (**B**). FTIR overlay display of (a) clomipramine, (b) crospovidone, and (c) their mixture; and (**C**). FTIR overlay display of (a) clomipramine, (b) *Plantago ovata* mucilage, and (c) their mixture.

FTIR studies were carried out to check drug–excipient compatibility. It was evident from the spectra that all three polymers did not interact with the drug [29,30,31], as can be seen in Figure 1A–C. The blue color peak (a) represents the drug (clomipramine), the red color peak (b) represents superdisintegrants, such as croscarmellose sodium (CS), crospovidone (CP), and *Plantago ovata* mucilage (POM), and the green color peak (c) represents the mixture of the drug and excipients. FTIR analysis of the drug showed specific characteristic bands linked to the quaternary ammonium group (–NH+–(CH_3_)_2_) which reflects three bands in the region of 2926–2450 cm^−1^. The –(CH_3_) (methylene group) attachment to the seven-membered ring nitrogen atom was seen at 2646 cm^−1^. The asymmetric stretching vibration band of C–N was seen at 1234 cm^−1^ and the chlorine atom attached to the aryl group was observed at 1105 to 940 cm^−1^. The FTIR spectrum of CS in Figure 1I peak (b) showed characteristic peaks at 3444 cm^−1^ to 3273 cm^−1^ linked to hydroxyl (O–H) groups and the peaks at 2895.15 cm^−1^ to 2420.66 cm^−1^ are linked to C–H (sp3) stretching vibrations. The carbonyl (C=O) group of carboxylic acid is represented by the presence of a peak at 1637.56 cm^−1^. The FTIR spectrum of CP in Figure 1II peak (b) showed characteristic peaks at 3466 cm^−1^ to 3292 cm^−1^ which are linked to hydroxyl (O–H) groups. The peaks at 2953.02 cm^−1^ to 2409.09 cm^−1^ are linked to C–H (sp3) stretching vibrations while the carbonyl (C=O) group of carboxylic acid is represented by the presence of a peak at 1660.71 cm^−1^. The FTIR spectrum of POM in Figure 1III peak (b) showed characteristic peaks at 3442 cm^−1^ and 3315 cm^−1^ which are linked to hydroxyl (O–H) groups. The peak at 2923.20 cm^−1^ is linked to C–H stretching vibrations, and the carbonyl (C=O) group of carboxylic acid is represented by the presence of a peak at 1631.78 cm^−1^. The FTIR analysis of a mixture of CLP (drug) and all three used superdisintegrants revealed the clear presence of drug functional groups but with carrier bands showing minor shifts. Overall, the superdisintegrants used had been found to be compatible with the drug (CLP) and were employed to develop the desired ODT formulations.

#### 2.2.2. Differential Scanning Calorimetry (DSC)/Thermogravimetric Analysis (TGA)

**Figure 2:**
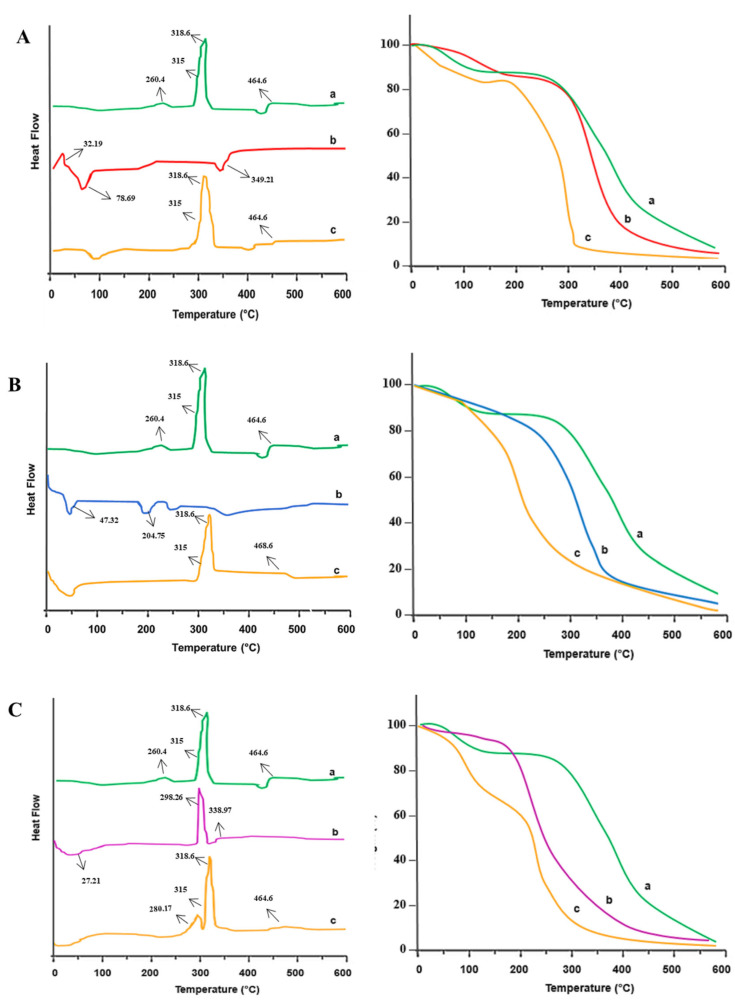
(**A**). DSC/TGA analysis of (a) clomipramine, (b) croscarmellose sodium, and (c) their mixture; (**B**). DSC/TGA analysis of (a) clomipramine, (b) crospovidone, and (c) their mixture; and (**C**). DSC/TGA analysis of (a) clomipramine, (b) *Plantago ovata* mucilage, and (c) their mixture.

DSC/TGA analyses were carried out to check the drug-excipients’ thermal compatibility. It was evident from the graphs that all used excipients did not interact with the drug (Figure 2A–C). The green color peak (a) represents the drug (CLP); peak (b) represents superdisintegrants, such as croscarmellose sodium (CS) in the red color, crospovidone (CP) in the blue color, and *Plantago ovata* mucilage (POM) in the purple color; and the mustard color peak (c) represents a mixture of the drug and excipients. The DSC thermogram of pure CLP showed exothermic peaks at 260.4 °C, 315 °C, 318.6 °C, and 464.6 °C, while an endothermic peak at 454.2 °C that corresponded to water absorption and evaporation [32]. The DSC thermogram of CS showed an exothermic peak at 32.19 °C and endothermic peaks at 78.69 °C and 349.21 °C linked to the decomposition of CS [33]. The DSC thermogram of CP showed endothermic peaks at 47.32°, 204.75 °C, and 309.5 °C linked to the evaporation of water trapped inside the polymeric network [33]. The DSC thermogram of POM showed an endothermic peak at 27.21 °C and a broad exothermic peak at 298.26 °C, indicating that a crystalline nature is present in POM [34]. Overall, the DSC thermogram of the physical mixture of the drug and superdisintegrants indicated no interaction, maintaining the actual nature of the drug (clomipramine).

#### 2.2.3. Powdered X-ray Diffraction (PXRD) Analysis

**Figure 3:**
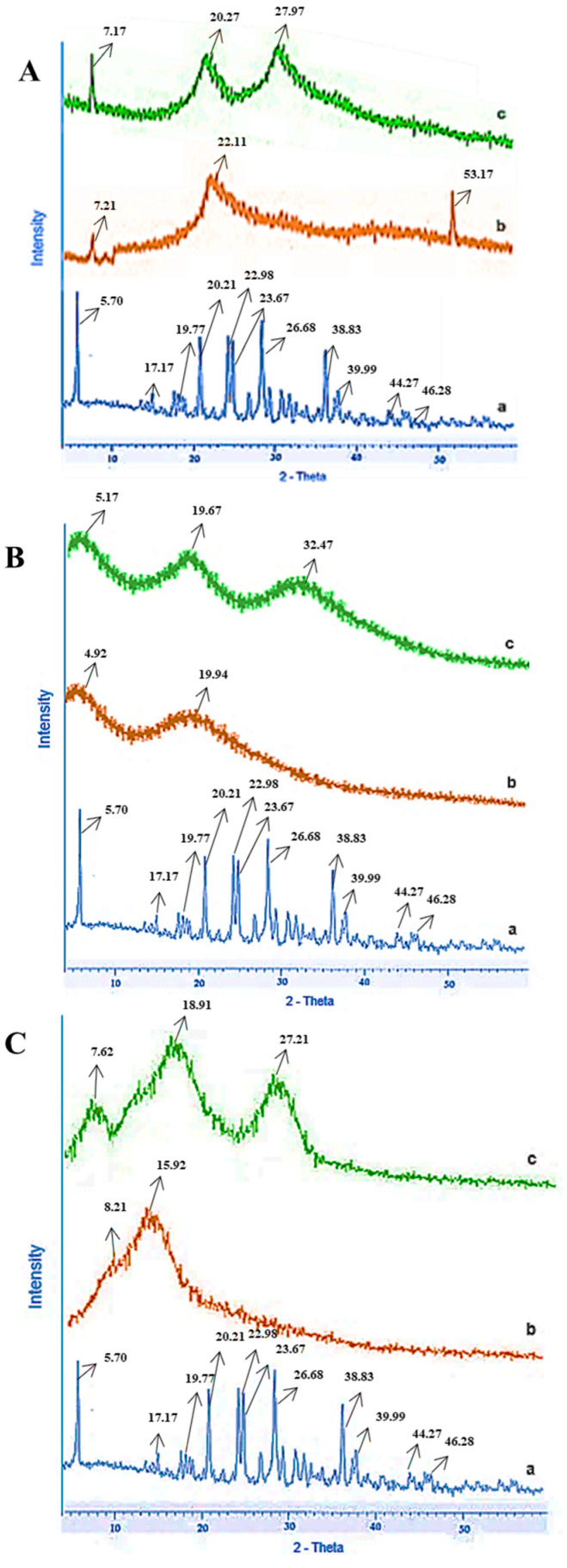
(**A**). PXRD analysis of (a) clomipramine, (b) croscarmellose sodium, and (c) their mixture; (**B**). PXRD analysis of (a) clomipramine, (b) crospovidone, and (c) their mixture; and (**C**). PXRD analysis of (a) clomipramine, (b) *Plantago ovata* mucilage, and (c) their mixture.

PXRD analysis was carried out to see the crystalline nature linked to the stability of the drug clomipramine (CLP). It was evident from the graphs that none of the excipients interacted with the drug, as seen in Figure 3A–C. The blue color peak (a) represents the drug (clomipramine); the red color peak (b) represents superdisintegrants such as croscarmellose sodium (CS), crospovidone (CP), and *Plantago ovata* mucilage (POM); and the green color peak (c) represents the mixture of drug and excipients. The PXRD analysis of clomipramine revealed a series of intense peaks at 5.70°, 17.17°, 19.77°, 20.21°, 22.98°, 23.67°, 26.68°, 38.83°, 39.99°, 44.27°, and 46.28° (2θ) that clearly represent the crystalline nature of clomipramine (CLP) [35]. The PXRD analysis of CS showed peaks at 7.21°, 22.11°, and 53.17° (2θ), highlighting the more amorphous and less crystalline nature of CS [36]. The PXRD analysis of CP showed peaks at 4.92° and 19.94° (2θ), also highlighting the amorphous nature of CP [37]. The PXRD analysis of POM showed a highly intense peak at 15.92° (2θ), highlighting the crystalline nature of POM [38]. Overall, the physical mixture of drug and superdisintegrants maintains the series of intense peaks linked to the crystalline nature of the drug, showing no interaction between the drug and excipients used.

### 2.3. Formulation Design and Optimization by Design Expert

The Central Composite Design (CCD) has become an essential step in drug designing for optimizing and enhancing the design of experiments with maximum practical proficiency. This analysis is very effective at overcoming the insufficiencies of classical optimization routes. During our present research work, 2-factor 2-level CCD was adopted to formulate and optimize the ODTs. Two independent factors such as superdisintegrant (CS/CP/POM) and diluent (avicel) concentrations were assessed quantitatively to evaluate their effect against dependent responses, i.e., % friability and disintegration time (s). An ANOVA (analysis of variance) was used to evaluate the significant effects of all factors on selected responses, i.e., % friability and disintegration time (s). The *p*-value was found to be significant, i.e., less than 0.1, predicting a substantial effect of concentrations of the synthetic disintegrant, CP = crospovidone, and natural superdisintegrant, POM = *Plantago ovata* mucilage, on friability and disintegration time. The ANOVA was not applied on CS as the model linked to CS:Avcl because the formulation was very weak and did not produce statistically significant results. The variables were fitted into various regression statistical models considering several other parameters such as correlation coefficient, R^2^ predicted, and adjusted R^2^ (Table 2). The most accurate model for each response was justified by Fisher value and prob values. The software suggested the quadratic model as the best-fit model with prob > F value and maximum R^2^ as seen in Table 3. The F values and *p*-values of the friability and disintegration time for all formulations, respectively, are noted in Table 3. The best-fit model with maximum R^2^ values was found for the natural superdisintegrant (POM:Avcl) formulations as compared to synthetic disintegrants (CS:Avcl and CP:Avcl). The predicted R^2^ of 0.8094 is in acceptable agreement with the adjusted R^2^ of 0.9459 for friability and the predicted R^2^ of 0.8424 is in acceptable agreement with the adjusted R^2^ of 0.9314 for disintegration time (i.e., the difference is less than 0.2). The signal-to-noise ratio was determined by adequate precision; a ratio greater than four is desirable. An adequate precision linked to friability and disintegration time indicates an adequate signal. The results observed for % friability and disintegration time (s) were 17.6655 and 16.2619, respectively. The final equations in terms of actual factors (measured values) are also mentioned in [24].

% Friability (R1) = 0.35 − 0.087X1 + 0.034X2 − 0.004X1X2 + 0.054X1^2^ + 0.009X2^2^

Disintegration time (R2) = 11.33 − 2.93X1 + 1.26X2 − 0.049X1X2 − 0.02X1^2^ + 2.17X2^2^

**Figure 4:**
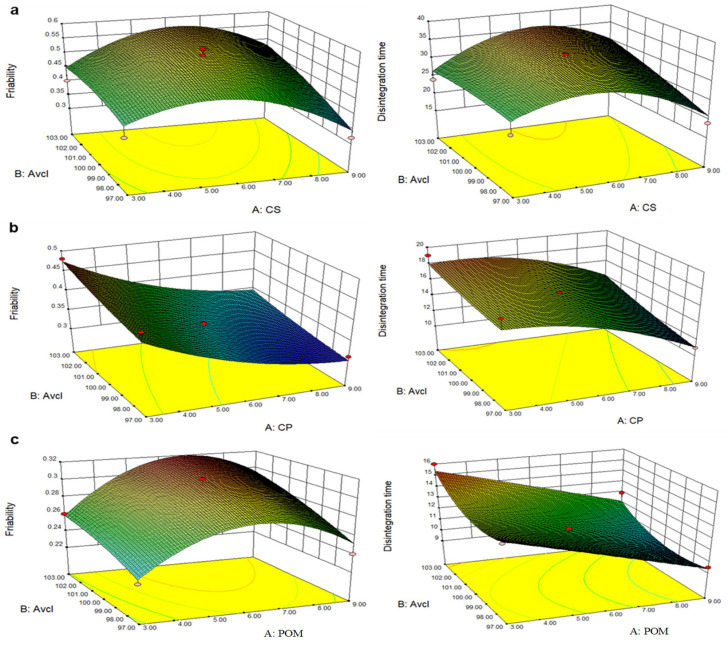
Three-dimensional response surface plots of friability (%) and disintegration time (s); (**a**) CS:Avcl; (**b**) CP:Avcl; and (**c**) POM:Avcl.

**Figure 5:**
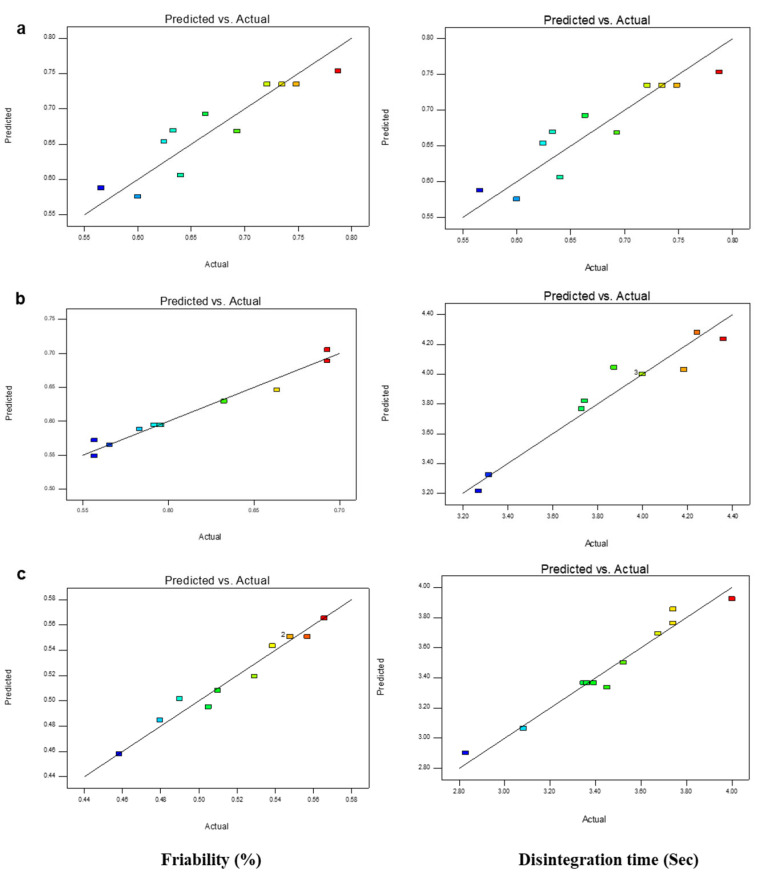
Plots of predicted vs. actual response of friability (%) and disintegration time (s); (**a**) CS:Avcl; (**b**) CP:Avcl; and (**c**) POM:Avcl.

**Figure 6:**
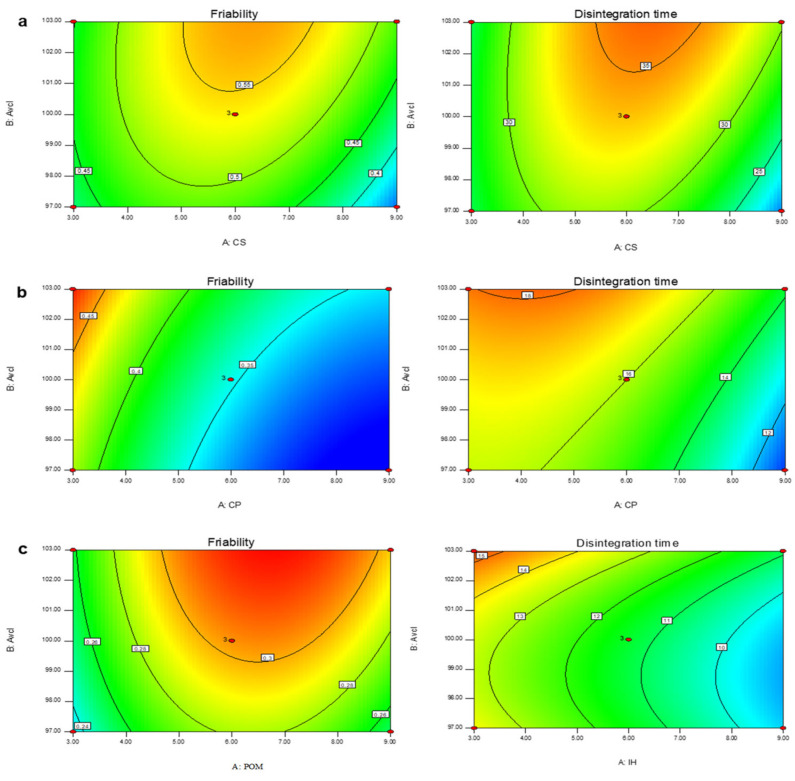
Two-dimensional contour plots of friability (%) and disintegration time (s); (**a**) CS:Avcl; (**b**) CP:Avcl; and (**c**) POM:Avcl.

In addition, the linear correlation graphs between the actual and predicted response values were found to be close on a straight line. With this high degree of fit, the data were considered accurate with an affirmation of model appropriateness for the simulation of experimental data for the ODTs formulations. Three different graphs (3D response surface, predicted vs., actual values, and 2D contour plots) were used for elaborating the effect of all three disintegrants (CS/CP/POM) and diluent (avicel) concentration on friability and disintegration time graphically (Figure 4, Figure 5 and Figure 6) [23,39]. The coefficient values of the polynomial equation were found to have a positive effect on % friability and disintegration time when the concentrations of the superdisintegrant fluctuated randomly between the highest and lowest levels. The ANOVA results confirmed that the % friability and disintegration are highly dependent on the concentration of superdisintegrant and diluent used [40].

The fit summary for the regression models and ANOVA results linked to POM:Avcl formulations are mentioned in Table 2 and Table 3, respectively, and the fit summary for the regression models linked to CS:Avcl and CP:Avcl formulations and the ANOVA results linked to CP:Avcl are mentioned in the Supplementary Data in Tables S1 and S2, respectively. The models of the CS:Avcl formulations seemed very weak so we did not obtain ANOVA results for them. To optimize the factors involved in drug designing to attain improved and desired formulation, the desirability factor is used. After primary analysis, the desired parameters were set to attain friability around 0.24% and a rapid disintegration time of fewer than 10 s. The optimized formulation was found to have a POM:Avcl ratio of 9:97 with the desired maximum R^2^ = 0.9730 (shown in Table 4).

### 2.4. Pre-Compression Evaluation of Powder Blend

The bulk densities of blends observed at the pre-compression stage for each batch ranged from 0.40 to 0.49 g/mL. The tapped densities were measured after tapping the mixture of powder a specific number of times and ranged within 0.45–0.59 g/mL. The calculated Carr’s index values were within the range of 7.69 to 18.36%. Hausner’s ratio also confirmed good flow characteristics as values ranged from 1.08 to 1.22. The values of the angle of repose were <30°, indicative of good powder flow [41].

**Figure 7:**
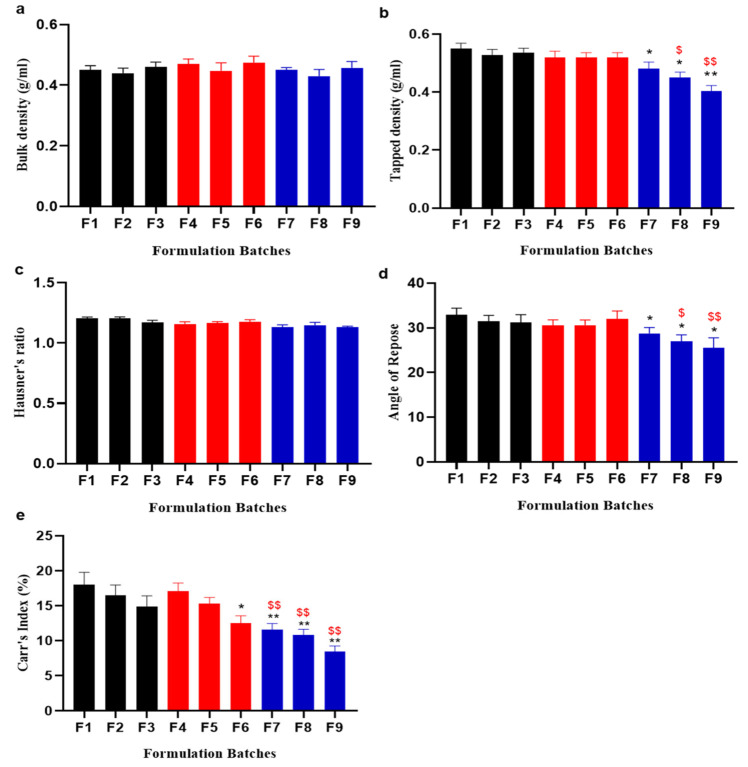
Comparison of pre-compression parameters: (**a**) Bulk density, (**b**) tapped density, (**c**) Hausner’s ratio, (**d**) angle of repose, and (**e**) Carr’s index of all prepared formulations with the two synthetic polymers, CS and CP, and one natural superdisintegrant, POM. F1–F3, represent the different ratios of CS:Avcl, F4–F6, represent the different ratios of CP:Avcl, and F7–F9, represent the different ratios of POM:Avcl. For all graphs, each column is expressed as the means ± SEM (*/^$^ *p* < 0.05; **/^$$^ *p* < 0.01; * is linked to statistically significant results on the comparison of F1–F3 to F4–F6 and F7–F9 at the same concentration of disintegrant and diluent used, and ^$^ is linked to statistically significant results on the comparison of F4–F3 to F7–F9 at the same concentration of disintegrant and diluent used.

In the present research, the effectiveness of the *Plantago ovata* mucilage as a superdisintegrant for orodispersible tablets was checked. Different pharmaceutical characteristics such as the density, Hausner’s ratio, and Carr’s index loss on drying was compared with the alternative superdisintegrants, croscarmellose sodium and crospovidone. ODTs quickly disintegrated because of the *Plantago ovata* mucilage’s high swelling index, which was highly concentration-dependent. Additionally, it improved the compressive performance of ODTs. Despite being insoluble in water, the mucilage from the *Plantago ovata* is able to absorb high levels of water and swells [22]. The direct compression method was used for ODT formulations of tricyclic antidepressant drugs with the use of *Plantago ovata* mucilage as a superdisintegrant in several concentrations. All the formulations showed a superb flow ability delineated in terms of the angle of repose. The bulk and tapped density showed an acceptably good range, which indicates that they have good packability. Carr’s index values and Hausner’s ratio explain that SDs had excellent compressibility and good flow properties [42], as shown in Figure 7.

### 2.5. Post-Compression Tablet Evaluation

Clomipramine tablets’ weight ranged from 135.6–122.9 mg, well within the acceptable 10% compendia range of 130 mg, with negligible batch-to-batch variations. The tablet thicknesses were measured and it was noted that all the tablets were consistent. The tablets’ hardness was adequate to abide shocks and yet had enough softness to cause abrupt fragmentation upon contact with water. The hardness noted was between 3.89 kg/cm^2^ and 4.87 kg/cm^2^, which were within the acceptable limits [43]. The experimental tablets’ mechanical strength was evaluated by measuring their friability. All nine batches of the tablets had observed friability that ranged from 0.23% to 0.51%. Drug content uniformity assay showed that the active ingredients ranged from 93.79% to 99.41% and were in the acceptable range of ±7.5%.

According to research, the use of a large amount of superdisintegrant results in tablets with poor friability. Therefore, a low compression force should be applied due to the poor friability of ODT formulations. The *Plantago ovata* mucilage overcomes this problem by enhancing the mechanical strength via powerful linkage formation within the particles of ingredients used to strengthen the ODT formulations. On the other hand, this natural superdisintegrant has the ability to absorb more water leading to speedy disintegration [44].

The water absorption ratio was checked and ranged from 54.94 to 66.7% and the wetting time was also checked and found within the range of 7.23–26.47 s, which was within the prescribed limits. Wetting time decreases and the absorption ratio increases by increasing the concentration of superdisintegrants. A larger decrease in wetting time was observed in POM-linked ODTs, which is further linked to the enhanced uptake of water by POM compared to CP and CS.

**Figure 8:**
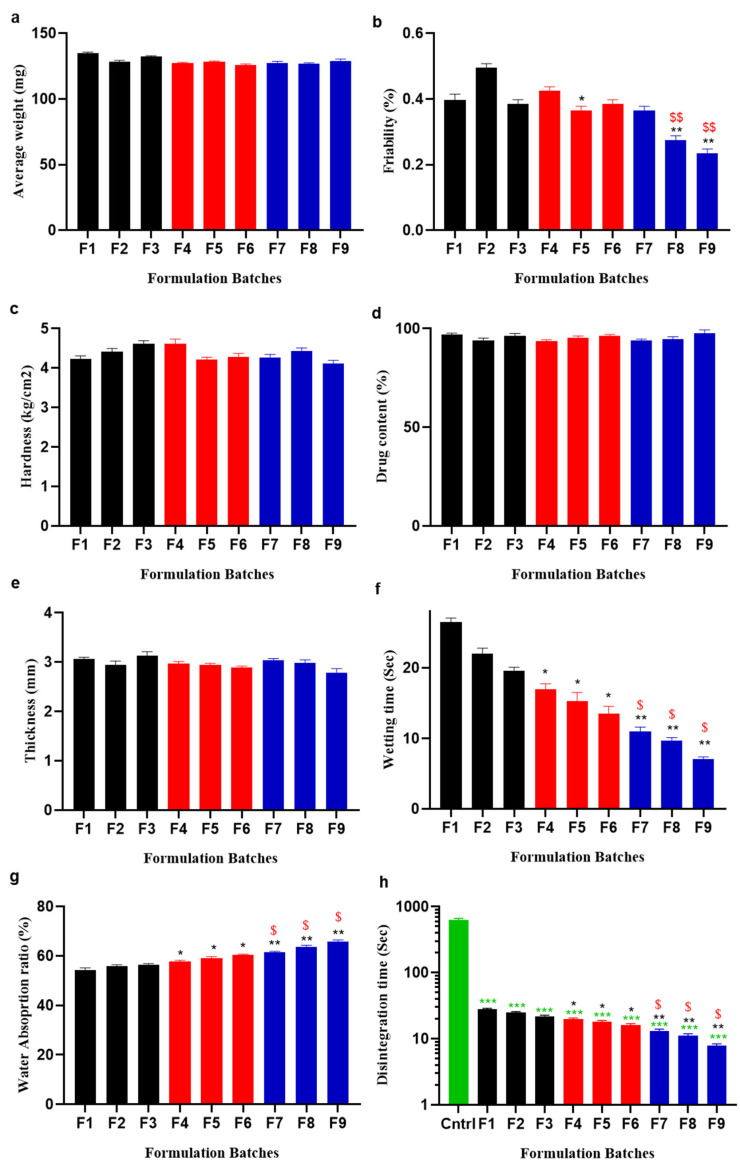
Comparison of post-compression parameters: (**a**) Average weight, (**b**) friability, (**c**) hardness, (**d**) drug content, (**e**) thickness, (**f**) wetting time, (**g**) water absorption ratio, and (**h**) disintegration time of all prepared formulations with the two synthetic polymers, CS and CP, and one natural superdisintegrant, POM. F1–F3, represent the different ratios of CS:Avcl, F4–F6, represent the different ratios of CP:Avcl, and F7–F9, represent the different ratios of POM:Avcl. For all graphs, each column is expressed as the means ± SEM (*/^$^ *p* < 0.05; **/^$$^ *p* < 0.01; *** *p* < 0.001),* is linked to statistically significant results on the comparison of F1–F3 to F4–F6 and F7–F9 at the same concentration of disintegrant and diluent used, and ^$^ is linked to statistically significant results on the comparison of F4–F3 to F7–F9 at the same concentration of disintegrant and diluent used. All prepared ODTs were also compared with the commercially available non-ODT product clomfranil as a control.

The in vitro disintegration time was measured individually for each SDs formulation to assess its fragmentation capabilities and compared with the commercially available clomfranil as a control. Ideal superdisintegrants should be able to induce tablet disintegration within short intervals of time, i.e., not exceeding 3 min, with no residue left. Results of the present work were very promising, i.e., tablets disintegrated within 29 s as described in Figure 8. All the prepared formulations, specifically those in which POM was used, showed speedy disintegration within the oral cavity. Croscarmellose sodium was incorporated in formulations F1, F2, and F3 in increasing order of concentration, i.e., 3, 6, and 9 mg/tablet. Disintegration time (D.T) was found to be connected directly with concentration of croscarmellose, i.e., D.T was lowered with the increase in croscarmellose concentration, while wetting time was also decreased. 

The superdisintegrant capability of crospovidone was checked in formulations F4, F5, and F6. All formulations disintegrated in less than one minute and there was a decreasing trend with the increasing concentration of crospovidone. *Plantago ovata* mucilage was incorporated in F7, F8, and F9 formulations similarly to the synthetic polymers. With the increase in *Plantago ovata* mucilage concentration, there was a decrease in DT, and the F9 formulation showed a DT of 8.39 sec and was selected as a desirable formulation. Overall, all the prepared formulations significantly fulfilled the criteria of ODTs with a rapid disintegration time as compared to the commercially available non-ODT clomfranil product. From all batches, one batch per superdisintegrant, F3 (CS), F6 (CP), and F9 (POM) were selected for in vitro drug release studies, due to their minimum disintegration time/superdisintegrant used, and compared with clomfranil as a control as shown in Figure 8.

### 2.6. In Vitro Drug Profiles

**Figure 9:**
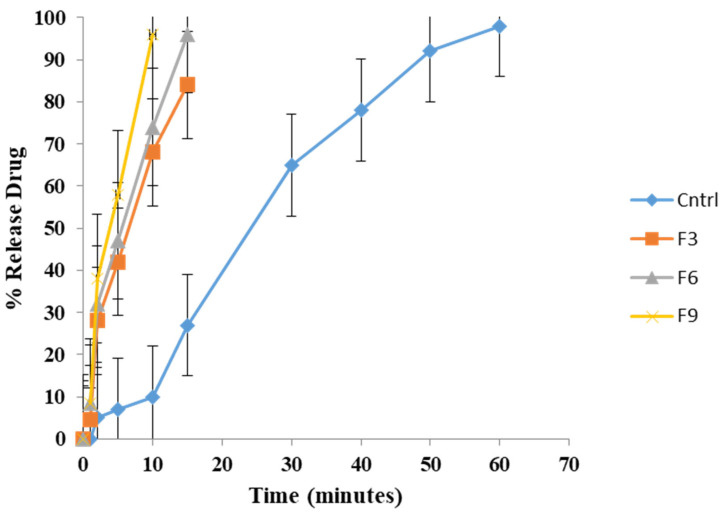
In vitro dissolution profile of clomfranil as a control and CLP ODTs. The drug release profile for the formulations (F3, F6, and F9) was noted as the mean of triplicates, with a phosphate buffer, pH 6.8, at 37 ± 0.5 °C.

Drug release studies were performed at the in vitro level on commercially available non-ODT clomfranil (used as a control) and formulations F3, F6, and F9 to compare the effect of three different SDs used and to determine the difference between the ODT and non-ODT; the results are shown in Figure 9. The in vitro drug release study of formulations F3, F6, and F9 revealed that most of the drug was released in a very short time, i.e., 9–15 min, whereas the formulation containing 9% *Plantago ovata* mucilage (F9) presented the fastest release and the whole drug was released in 9 min. In general, all the formulations showed a dissolution rate in the given order: F_3_ > F_6_ > F_9_ > Cntrl; the ODT drug dissolution is proportional to the time it takes for them to disintegrate [16,45]. Overall, all of the prepared formulations significantly fulfilled the criteria of ODTs with a rapid dissolution rate as compared to the commercially available non-ODT product, clomfranil. The formulation F9 containing 9 mg of POM showed the fastest dissolution rate as compared to other formulations in which a synthetic superdisintegrant at an equal concentration level as that of POM was used. Therefore, batch F9 was perceived as an optimized formulation.

### 2.7. In Vivo Study of Antidepressant Activity of Optimized Formulation (F9)

**Figure 10:**
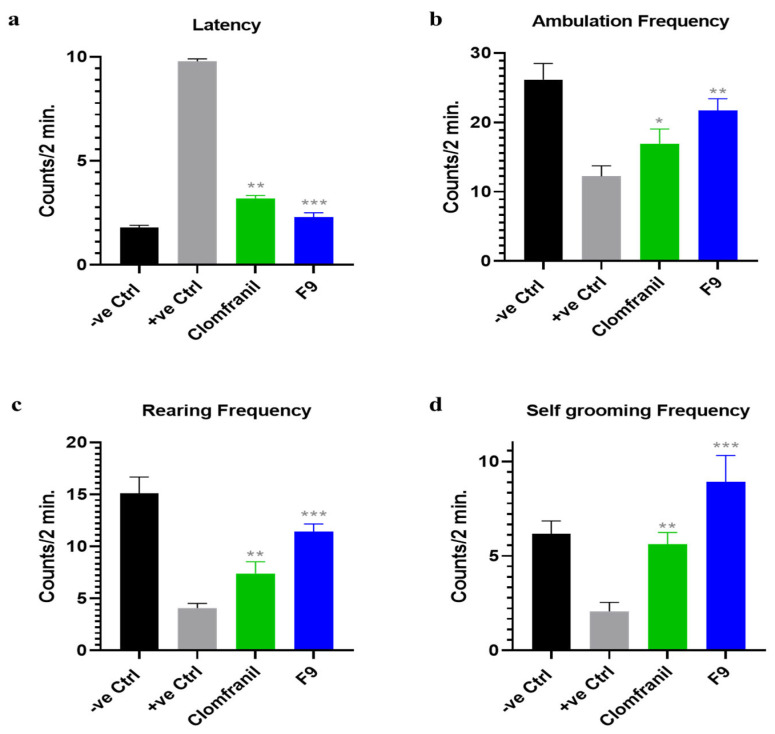
The anti-depressant effect of the optimized formulation (F9) and commercially available clomfranil tablet on the depressed behavior of rats by applying OFT. (**a**) Latency, (**b**) ambulation frequency, (**c**) rearing frequency, and (**d**) self-grooming frequency. For all graphs, each column is expressed as the means ± SEM (* *p* < 0.05; ** *p* < 0.01; *** *p* < 0.001).

**Figure 11:**
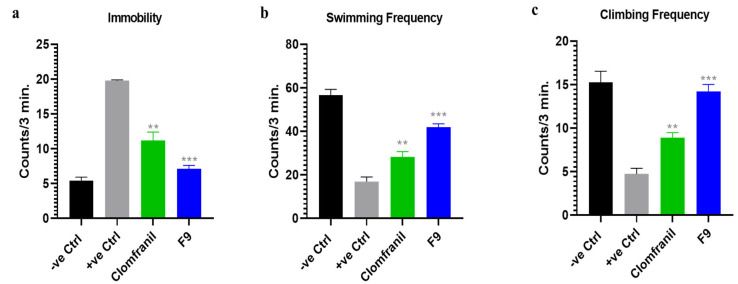
The anti-depressant effect of optimized formulation (F9), and the commercially available clomfranil tablet on the behavior of rats by applying the FST. (**a**) Immobility, (**b**) swimming frequency, and (**c**) climbing frequency. For all graphs, each column is expressed as the means ± SEM (** *p* < 0.01; *** *p* < 0.001).

The anti-depressant efficacy of the optimized ODT made by using POM (F9) was checked by an open-field test (OFT) of behavioral patterns, such as activities linked to exploration behavior, and hopelessness and despair behavior were checked as a model of depression in animals (albino rats) by applying the forced swimming test (FST). The behavioral effects of the optimized formulation (F9) after administration were compared with clomfranil tablets, a standard and approved antidepressant marketed in Pakistan. The antidepressant activity analysis results are shown in Figure 10 and Figure 11.

#### 2.7.1. Exploration Behavior Analysis via OFT

- *Latency*: Clomfranil decreased the latency in Group III linked depressed rats to 3.2, whereas the optimized formulation (F9) decreased the latency frequency more than clomfranil, due to its rapid action, to 2.28 in Group IV, as compared to the depressed rats of Group II that were kept without (F9) treatment.
- *Ambulation frequency*: An increase of up to 16.9 in the ambulation score was seen by using clomfranil as compared to Group II (depressed rats). On the other hand, the optimized formulation (F9) showed a larger increase in the ambulation score, 21.7, compared to Group II and Group III.
- *Rearing frequency*: Similar to ambulation, an increased rearing score of up to 7.4 was seen in Group III after treatment with clomfranil as compared to Group II, without clomfranil treatment. However, the optimized formulation (F9), showed a higher raise in the rearing score of 11.4 in Group IV as compared to Group II and Group III.
- *Self-grooming frequency*: After the ambulation and rearing score, an increase in self-grooming was seen up to 5.6 in Group III after treatment with clomfranil, as compared to Group II without clomfranil treatment. On the other hand, the optimized formulation (F9) showed a larger increase in the self-grooming score of 8.9 in Group IV as compared to Group II without (F9) and Group III with clomfranil treatment.

#### 2.7.2. Hopelessness and Despair Behavior Analysis via FST

- *Immobility frequency:* Clomfranil decreased the immobility in Group III linked depressed rats to 11.2, whereas the optimized formulation (F9) decreased the immobility frequency more than clomfranil due to its rapid action and was 7.2 in Group IV as compared to the depressed rats of Group II that were kept without (F9) treatment.
- *Swimming frequency:* An increase of up to 28.82 in the swimming frequency was seen by using clomfranil as compared to Group II (depressed rats). On the other hand, the optimized formulation (F9) showed a larger increase in the swimming frequency, 41.9, as compared to Group II and Group III.
- *Climbing frequency:* After swimming, an increase of 8.2 in the climbing frequency was also seen by using clomfranil as compared to Group II (depressed rats). However, the optimized formulation (F9) showed a larger increase in the climbing score, 14.2, as compared to Group II and Group III.

This in vivo study of anti-depressant activity revealed that the efficiency of the selected optimized formulation (F9) in the treatment of depression is more than the available clomfranil tablet, and may be linked to the rapid disintegration and bypassing of the systemic portal circulation.

## 3. Materials and Methods

### 3.1. Materials

Clomipramine (Glitz Pharma Pvt. Ltd., Islamabad, Pakistan), crospovidone (Medizan Pharma Pvt, Ltd., Islamabad, Pakistan), croscarmellose sodium (Caraway Pharma Pvt. Ltd., Rawalpindi, Pakistan) were purchased for use. *Plantago ovata* seeds were purchased from the local market of Sargodha, Pakistan. Magnesium stearate was acquired from Merck Pvt. Ltd., Karachi, Sind, Pakistan, aspartame was procured from Uni-Chem Labs Pvt. Ltd, Lahore, Punjab, Pakistan, and avicel was purchased from Relizon Pharma Pvt. Ltd., Lahore, Punjab, Pakistan. Reverse osmosis was used to obtain pure water. All the remaining chemicals used in formulations were of analytical grade.

### 3.2. Methods

#### 3.2.1. Extraction/Purification of *Plantago ovata* Mucilage (POM)

The powdered *Plantago ovata* seeds were ground in an ultra-centrifugal mill for 5 min (RetschTM ZM 200 Model; Fisher Scientific, Loughborough, UK) using a Retsch FV 2703 rotor. The ground powder was separated using a 0.5 mm sieve set [22]. The powder that had been sieved was gathered and kept for future use in an airtight, dry container. The POM was obtained by blending *Plantago ovata* powder along with water, following the ratio of 1:100 (*w*/*v*) with continuous stirring for 1.2 h at 80 °C. The extract was stored at 4 °C for subsequent use after being filtered using gauze to separate the mucilage. The POM was agitated in ethanol at a 100:75 proportion for 10 min (mucilage volume:ethanol volume). Following agitation, the samples were centrifuged at 37 °C for 3 min at around 2000 rpm. A rotary extractor was used to extract the ethanol from the precipitate. The spray drying technique was used for drying the samples [46]. After drying, the samples were stored in a well-closed container for further use.

#### 3.2.2. Physicochemical Characterization of Superdisintegrants

Physicochemical characterization of the superdisintegrants *Plantago ovata* mucilage, crospovidone, and croscarmellose sodium, such as the swelling ratio, weight loss on drying, bulk density, tapped density, Carr’s index, Hausner’s ratio and angle of repose, was determined.

##### Swelling Ratio

The superdisintegrants were placed individually in 100 mL graduated cylinders; each one was taken in a quantity of 1 g. The volume was made up to 100 mL by adding water. The cylinder was agitated vigorously at intervals of 10 min for one hour in order to obtain uniform distribution of the superdisintegrant. It was then left in an upright position for 24 h. The volume of sediment was calculated after 24 h [19].

$$Swelling\;ratio=\frac{volume\;after\;swelling}{volume\;before\;swelling}$$

##### Weight Loss on Drying

To investigate the quantity of the volatile materials or water, the loss on drying procedure was applied. Loss on drying was determined by taking an adequate amount of the powder, thoroughly mixed and weighed accurately, and placing it in a Petri dish at 105 °C for two hours in a 28-hot air oven. The weight of the powder was measured until the weight was a constant value [47]. The following formula was applied to calculate % LOD.

$$\%\;LOD=\frac{weight\;of\;water\;in\;sample}{weight\;of\;dry\;sample}\times 100$$

#### 3.2.3. Pre-Compression Parameters of the Powder Blend

All the materials were sieved with a no. 60 sieve (Dual Manufacturing Co., Franklin Park, IL, USA). For each given formulation, the appropriate amount of each substance was taken. The compressibility factor of the powder mixture was then assessed.

##### Determination of Bulk Density, Tapped Density

A weighed quantity of (50 g) was added into a graduated cylinder and the bulk density was found by measuring the volume occupied by powder. Then, the cylinder containing a known amount of powder was subjected to tapping for about 1 min on a balanced surface until a constant volume was obtained [19].

$$Bulk\;density=\frac{Weight\;of\;Powder\;}{Volume\;of\;powder\;before\;tapping\;}$$

$$Tapped\;density=\frac{Weight\;of\;Powder\;}{Volume\;of\;powder\;after\;tapping}$$

##### Angle of Repose

A HMKFlow 316 aluminum oxide angle of repose tester was used with a diameter of platform 22.5 cm. The height and radius of the formed cone were measured, and the angle of repose was calculated using the following equation.

Tan θ = *h/r*

where *h* and *r* are the height and radius of the powder cone, respectively.

For good flowing properties, the angle of repose should be less than 30°.

##### Powder Compressibility

To measure the compressibility of the powder, Carr’s index and Hausner’s ratio were calculated using the following formulas.

$$Carr’s\;index=\frac{Tapped\;density-Bulk\;density}{Tapped\;density}\times 100$$

$$Hausner’s\;ratio=\frac{Tapped\;density}{Bulk\;density}$$

#### 3.2.4. Drug-Superdisintegrants Compatibility Study

A suitable formulation is a key factor in the development of any dosage form. Physical or chemical compatibility was determined using Fourier Transformed Infrared Spectroscopy (FTIR), Differential Scanning Calorimetry (DSC)/Thermogravimetric Analysis (TGA), and X-ray diffraction (XRD) analysis.

##### Fourier Transform Infrared Spectroscopy (FTIR) Analysis

An FTIR spectroscophotometer (IR-Prestige21 Shimadzu) at the resolution of 2 cm^−1^ with a scanning range of 4000–400 cm^−1^ was used for functional group recording. This technique allows for predicting any type of incompatibility among different ingredients used in the formulation. The drug and all remaining excipients were mixed in a ratio of 1:100 with KBr to form a pellet and then combined in a small mortar and pestle before recording results with the FTIR under a pressure of 65 kN for 120 s. CLP, CS, CP, and POM spectra were taken separately and then with the drug [48].

##### Differential Scanning Calorimetry (DSC) and Thermogravimetric Analysis (TGA)

Thermal analysis of the drug (CLP) and superdisintegrants (CS, CP, and POM) was conducted using a DSC/TGA analyzer (DSC-60, Shimadzu, Kyoto, Japan and TGA, NETZSCH Company, Waldkraiburg, Bavarian State, Germany) separately and then combined. The thermal behavior of samples was analyzed at a temperature range of 0 to 600 °C at the rate of 10 °C/min under a nitrogen stream [49].

##### Powdered X-ray Diffraction (PXRD) Analysis

A powdered X-ray diffraction (XRD) analysis was conducted to study the structure of the drug (CLP) and superdisintegrants (CS, CP, and POM) separately and then combined, using an X Shimadzu diffractometer 6100 with Ni-filtered CuKα radiation. The wavelength used was 1.314 Å at 25 °C with a voltage of 40 kV over 2° to 60° diffraction scanning range (2θ) [48].

#### 3.2.5. Formulation Design and Optimization by Design Expert

A 2-level 2-factor central composite design was used for optimizing our prepared ODT formulations. The preliminary trials and already published literature were used for the selection of independent factors and two dependent responses. An 11 runs-based drug design was formulated by a Software Design Expert (version 8.0.6, Minneapolis, MN, USA). The concentration of superdisintegrant (X1) CS = croscarmellose sodium, CP = crospovidone, and POM = *Plantago ovata* mucilage, and diluent (X2) Avcl = avicel were chosen as independent variables with the friability (R1) and disintegration time (R2) as dependent parameters (Table 5). All the other factors such as Mgst = Magnesium stearate, SSG = sodium starch glycolate, and Aspt = Aspartame were kept constant. The optimization of dependent responses was carried out by using response surface methodology (RSM). All the formulations were prepared thrice. The mean value of each formulation was fit into several mathematical models such as the cubic, linear, quadratic, and 2FI models. The fit summary generated by the software was used to select the best model on the basis of adjusted R^2^ and predicted R^2^, *p*-value, and residual sum of squares. The model in which the predicted correlation coefficient was closest to the adjusted correlation coefficient with a minimum *p*-value ≤ 0.2 was selected. A generalized quadratic equation was used to evaluate the relationship between factors and responses.

Y = b_o_ + b_1×1_ + b_2_X_2_ + b_11_X^1^_2_ + b_22_X^2^_2_ + b_12_X_1_X_2_

where Y = response; X_1_ = concentration of one of selected disintegrant used (CS/CP/POM) concentration; X_2_ = Avcl concentration; b_o_ = intercept; b_1_–b_5_ = coefficients; X^1^_2_/X^2^_2_ = second order effects of disintegrant and Avcl concentrations, respectively; and X_1_X_2_ = interactive effects of disintegrant and Avcl concentrations.

Moreover, the desirability parameter was utilized to optimize the data to obtain the formulation with the most optimal responses. Further validation of the statistical model was carried out by ANOVA before additional analysis [50,51].

##### Preparation of CLP ODTs

All of the ingredients as described in Table 6 have been passed via sieve #60 separately. Then, with the exception of magnesium stearate, all the ingredients were blended in a polythene bag for 5 min. Afterward, the blend was lubricated with magnesium stearate for three minutes. Subsequently, using a CADMACH SMS25 single punch tablet machine with a specified force applied of 400 kgf, the blend was compressed into tablets. The average tablet weighed 130 mg and had a diameter of 9 mm^2^.

#### 3.2.6. Evaluation of Orodispersible Tablets

##### Tablet Hardness

Using a Monsanto hardness tester (Pharma Test Germany), the hardness of all formulations was measured and expressed in kg/cm^2^. The hardness test was performed on three tablets, chosen randomly, by placing them between digital hardness tester arms [52].

##### Thickness and Diameter

To measure the thickness and diameter, a randomly selected tablet from each formulation was checked by a vernier caliper for thickness. Five measurements were taken and then the average was recorded. The diameter varied by ±5% up to 12.5 mm and by ±3% up to 15 mm [52].

##### Weight Variation

To perform this test, 20 tablets were selected from each formulation and weighed on an electrical balance (Model No. BL 220H; Shimadzu, Japan). Tablets were weighed individually and their reading was noted; the total weight of 20 tablets was noted cumulatively and the average weight was computed [53].

##### Friability

Ten tablets were taken randomly, weighed, and placed in the plastic chamber of an USP type friabilator USP (Roche, UK). The plastic chamber rotation rate was set at 25 rpm and the time for rotation was 4 min (20). The weight of the tablets was noted after de-dusting and the percentage friability was calculated by using the following formula [53].

$$x=\frac{w_{1}-w_{2}\;}{w_{1}\;}\times 100$$

where x represents percent weight loss, $w_{1}$ is matrices’ weight before the friability test, and $w_{2}$ is the matrices’ weight after the friability test.

##### Drug Content

From each batch, five tablets were randomly selected, and the average weight was calculated. Then, each tablet was ground to a fine powder and placed in a 100 mL volumetric flask. Phosphate buffer (pH 6.8) was used to dissolve the drug. The filtered solution was used and analyzed spectrophotometrically at a 252 nm wavelength using a UV-Visible spectrophotometer (Shimadzu—JAPAN, UV-1700 Pharmaspec) [54].

##### Water Absorption Ratio

For evaluation of the absorption ratio, three tablets per batch of superdisintegrants were chosen randomly and placed between layers of absorbent paper that were already fitted into a Petri dish. After this, the fitted paper was wet completely with distilled water. The starting weight of the tablet was marked as (Wb), which was actually the weight of the tablet before placing it in the Petri dish, and the wet tablet was reweighed and marked as (Wa) [22]. The water absorption ratio R was calculated using the below-mentioned equation.

R=100×Wa−Wb/Wa

##### Wetting Time

A small petri dish was taken and 6 mL of an aqueous solution containing methylene blue (dye) was placed in it. Then, a piece of double tissue paper was placed in it. A selected tablet from each formulation was positioned on the surface of the paper carefully and the wetting time was taken as the time that was required for the dye to reach the upper surface of the tablet. A time limit of 150 s was used; a higher value than this was considered inappropriate for the prepared ODT formulation [22,54].

##### Disintegration Time

Three tablets per batch were tested for disintegration time using a simplified disintegration procedure. For this experiment, a Petri plate with 10 mL of water maintained at a temperature of 37 ± 5 °C was used. The time for each tablet completely break into tiny particles after being carefully placed in the center of the petri dish was recorded [44].

##### In Vitro Drug Release

The in vitro USP paddle equipment type II (Pharma Test Germany) was used for drug release evaluation. The phosphate buffer, pH 6.8, was first diluted to 900 mL in a jar which was kept at a constant 37 ± 0.5 °C. The paddle rotated at a constant speed of 50 rpm. At various intervals of 3, 6, 9, 12, 15, 18, 21, and 24 min, samples (5 mL) were taken out and refilled with an equivalent volume of fresh fluid. After filtering, each sample’s absorbance was assessed using a UV spectrophotometer double beam (Shimadzu 1601, Japan) at a wavelength of 252 nm. The total percentage of the drug dissolved was used to compute the amount of the drug using the standard calibration curve [45].

#### 3.2.7. In Vivo Study of Antidepressant Activity of Optimized Formulation (F9)

To analyze the anti-depressant efficacy of the optimized ODT made by using POM (F9), behavioral patterns such as hopelessness and despair behavior were checked as a model of depression in animals (rats) by applying the forced swimming test (FST), and general activities linked to exploration behavior were checked by open-field test (OFT). The behavioral effects of the optimized formulation (F9), after administration, were compared with the clomfranil tablet (manufactured by Novartis), which is used as an approved and standard antidepressant marketed in Pakistan.

Animals (Swiss albino rats), weighing 100–120 g, were obtained from the animal facility of the University of Sargodha, Sargodha, Pakistan. All animals (equal number of males and females; *n* = 6) were divided into four main groups. Group I: negative control group (normal), Group II: positive control group (animals exposed to a depressive environment), Group III: depressed animals treated with clomfranil, and Group IV: depressed animals treated with F9. Clomfranil and F9 (10 mg/Kg) were given to allotted groups by administering the tablets through the oral cavity with the help of tweezers of a suitable size in accordance with the weight of the animal and keeping the animal in a static position by holding the mouth for few seconds until complete tablet disintegration [55,56]. Depression was induced by using the social isolation model. All the animals in Group II to Group IV were subjected to a depression model by housing them in the isolation chamber singly. Temperature, humidity, food, and water supply were maintained. This exposure was continuous for up to 21 days. After the trial period, Group III and Group IV were treated with clomfranil and F9, respectively, once on a daily basis for up to 14 days. At the end of drug treatment, the animals were subjected to exploration and depressed behavioral analysis via FST and OFT individually.

##### Exploration Behavior Analysis via OFT

The following were the noted parameters.

- *Latency*: This is linked to the time taken by the animal to decide to move after placing the animal in a beaker’s center.
- *Ambulation frequency*: This is linked to the mobility of the animal inside the beaker, e.g., the number of spherical movements of the animal made around the wall of the beaker.
- *Rearing frequency*: This means how many times the animal stood and stretched on its hind limbs without its forelimb support.
- *Grooming frequency*: This means how many times did the animal perform face touching/face scratching with the hind legs or how many times the animal licked its fur/genitals.

##### Hopelessness and Despair Behavior Analysis via FST

The following were the noted parameters [56].

*The latency of immobility*: is linked to the time when the animal was not engaged in active swimming, which means a total number of stops during swimming. When the animal remained floating in the water without struggling and with fewer movements of its limbs to keep their head above the water level, it will be considered immobile or depressed.

*Swimming*: active or normal animals will show enhanced forepaw movements throughout the beaker that indicate that the animal is trying to keep their head above the water.

*Climbing*: is linked to that activity wherein the animals show vigorous movements in and out of the water with their forepaws against the beaker’s wall.

#### 3.2.8. Statistical Analysis

Collected data for all the formulations were presented as the mean ± SD. One-way analysis of variance (ANOVA) and, for comparison, Tukey’s test was also applied in GraphPad Prism 8.2.1 software. Values of (* *p* < 0.05 and ** *p* < 0.01) were linked to statistically significant results of the comparison.

## 4. Conclusions

It was concluded that the prepared CLP ODTs showed good efficacy in antidepressant activity as compared with the commercially available dosage form. The main factor linked to ODTs, such as friability and disintegration time for all formulations, were successfully modeled and optimized using a central composite design. Using linear regression and surface plots, the technique has been found to be successful in evaluating the impact of factors on the response parameters. The responsive parameters were improved both numerically and graphically, and the projected optimum results were validated and proven to have been in excellent accordance with the projected results to within 5%. The order of rapid drug release based on superdisintegrants used in the above study was as follows POM > CP > CS. The in vivo study showed that the selected (F9) ODT has very good effectiveness in the treatment of depression.

## Figures and Tables

**Table 1:** The physical characteristic of superdisintegrants.

Parameter	POM	CS	CP
Angle of repose	30	28	29
Bulk density (g/mL)	0.569	0.55	0.52
Tapped density (g/mL)	0.590	0.66	0.63
Weight loss on drying (%)	7.1	9.5	13
Swelling index (%)	95.94	87	94
Hausner’s ratio	1.03	1.2	1.21
Carr’s index	5.08	16.67	17.46

POM: *Plantago ovata* mucilage; CS: Croscarmellose Sodium; and CP: Crospovidone.

**Table 2:** Fit summary for regression models (POM: Avcl Formulations).

POM: Avcl Formulations					
**Friability (%)**					
Source	Sequential *p*-value	Lack of Fit *p*-value	Adjusted R^2^	Predicted R^2^	Remarks
Linear	0.2532	0.0190	0.1133	0.3839	
2FI	0.8002	0.0160	0.0034	0.7181	
Quadratic	0.0006	0.1799	0.9459	0.8094	Suggested
Cubic	0.4426	0.1130	0.9292	0.0790	Aliased
Disintegration Time (s)					
Linear	0.0013	0.0152	0.7640	0.5975	
2FI	0.9057	0.0127	0.7309	0.4645	
Quadratic	0.0170	0.0389	0.9314	0.8424	Suggested
Cubic	0.3195	0.0283	0.9425	0.0438	Aliased

**Table 3:** ANOVA results for second polynomial equations (POM: Avcl Formulations); Response 1: R1 (Friability) and Response 2: R2 (Disintegration).

Response 1: R1 (Friability)						
**Source**	Sum of Squares	df	Mean Square	F-Value	*p*-Value	
**Model**	0.0399	5	0.0080	35.98	0.0006	significant
**A-POM**	0.0308	1	0.0308	138.69	<0.0001	
**B-Avcl**	0.0049	1	0.0049	21.91	0.0054	
**AB**	0.0000	1	0.0000	0.1126	0.7508	
**A^2^**	0.0042	1	0.0042	18.90	0.0074	
**B^2^**	0.0001	1	0.0001	0.5741	0.4828	
**Residual**	0.0001	5	0.0001			
**Lack of Fit**	0.0001	3	0.0001	0.6903	0.5806	not significant
**Pure Error**	0.0000	2	0.0000			
**Cor Total**	0.0410	10				
	**Fit Statistics**					
**Std. Dev.**		0.0149	**R^2^**		0.9730	
**Mean**		0.3763	**Adjusted R^2^**		0.9459	
**C.V. %**		3.96	**Predicted R^2^**		0.8094	
			**Adeq. Precision**		17.6655	
**Response 2: R2 (Disintegration time)**						
**Source**	Sum of Squares	df	Mean Square	F-Value	*p*-Value	
**Model**	48.18	5	9.64	28.15	0.0011	significant
**A-POM**	34.38	1	34.38	100.45	0.0002	
**B-Avcl**	6.41	1	6.41	18.74	0.0075	
**AB**	0.0025	1	0.0025	0.0073	0.9352	
**A^2^**	0.0006	1	0.0006	0.0018	0.9679	
**B^2^**	6.71	1	6.71	19.60	0.0068	
**Residual**	0.05	5	0.0005			
**Lack of Fit**	0.0002	3	0.0002	0.0007	0.4066	not significant
**Pure Error**	0.0467	2	0.0233			
**Cor Total**	47.86	10				
	**Fit Statistics**					
**Std. Dev.**		0.5851	**R^2^**		0.9657	
**Mean**		12.12	**Adjusted R^2^**		0.9314	
**C.V. %**		4.83	**Predicted R^2^**		0.8424	
			**Adeq. Precision**		16.2619	

**Table 4:** Optimized formulation with the experimental value of variables.

No	POM	Avcl	Friability (%)	Disintegration Time (s)	Desirability	
1	9.00	97.00	0.24	9.5	0.9730	Selected

**Table 5:** The central composite design of two independent factors against two dependent response variables.

No. of Run	Factor 1 (X1)	Factor 2 (X2)	Response 1 (R1)	Response 2 (R2)
CS:Avcl Formulations				
	X1: CS	X2: Avcl	R1: Friability	R2: Disintegration time
	Mg	Mg	%	Sec
1	3.00	97.00	0.52	34
2	6.00	100.00	0.62	38
3	9.00	97.00	0.39	23
4	6.00	100.00	0.36	21
5	6.00	104.24	0.54	33.9
6	6.00	95.76	0.56	33.8
7	3.00	103.00	0.401	23.9
8	9.00	103.00	0.32	19
9	1.76	100.00	0.41	24
10	6.00	100.00	0.44	29
11	10.24	100.00	0.48	32
CP: Avcl Formulations				
	X1: CP	X2: Avcl	R1: Friability	R2: Disintegration time
	Mg	Mg	%	Sec
1	3.00	97.00	0.44	17.5
2	6.00	100.00	0.35	16
3	9.00	97.00	0.31	11
4	6.00	100.00	0.355	16
5	6.00	104.24	0.4	18
6	6.00	95.76	0.31	14
7	3.00	103.00	0.48	19
8	9.00	103.00	0.34	13.9
9	1.76	100.00	0.48	15
10	6.00	100.00	0.354	16
11	10.24	100.00	0.32	10.7
POM:Avcl Formulations				
	X1: POM	X2: Avcl	R1: Friability	R2: Disintegration time
	Mg	Mg	%	s
1	3.00	97.00	0.23	13.5
2	6.00	100.00	0.3	11.5
3	9.00	97.00	0.24	9.5
4	6.00	100.00	0.31	11.2
5	6.00	104.24	0.32	14
6	6.00	95.76	0.28	12.4
7	3.00	103.00	0.26	16
8	9.00	103.00	0.29	11.9
9	1.76	100.00	0.21	14
10	6.00	100.00	0.3	11.3
11	10.24	100.00	0.255	8

**Table 6:** The final design of all formulations of CLP ODTs.

Composition	F_1_ (mg)	F_2_ (mg)	F_3_ (mg)	F_4_ (mg)	F_5_ (mg)	F_6_ (mg)	F_7_ (mg)	F_8_ (mg)	F_9_ (mg)
CLP CS	10 3	10 6	10 9	10 -	10 -	10 -	10 -	10 -	10 -
CP	-	-	-	3	6	9	-	-	-
POM	-	-	-	-	-	-	3	6	9
Avcl Mgst SSG Aspt	103 4 2 3	100 4 2 3	97 4 2 3	103 4 2 3	100 4 2 3	97 4 2 3	103 4 2 3	100 4 2 3	97 4 2 3

CLP = clomipramine, CS = croscarmellose sodium, CP = crospovidone, POM = *Plantago ovata* mucilage, Avcl = avicel, Mgst = Magnesium stearate, SSG = sodium starch glycolate, and Aspt = aspartame.

## Data Availability

Data is contained within the article and supplementary material.

## Supplementary Materials

The following supporting information can be downloaded at: https://www.mdpi.com/article/10.3390/ph16020265/s1, Table S1: Fit summary for regression models. Table S2: ANOVA results for second polynomial equations (CP: Avcl).
