# Supplementary material for: In Vitro/In Vivo Evaluation of Clomipramine Orodispersible Tablets for the Treatment of Depression and Obsessive-Compulsive Disorder
Source: Pharmaceuticals (Basel). 2023 Feb 9;16(2):265. doi: 10.3390/ph16020265 (PMC9967651; doi:10.3390/ph16020265)
Supplement: Supplementary file 1 [file pharmaceuticals-16-00265-s001.zip › pharmaceuticals-2116545-supplementary.pdf]

Supplementary data:

Table S1 Fit summary for regression models

CS : Avcl Formulations					
Friability (%)					
Source	*Sequential p-value*	Lack of Fit p-value	Adjusted R^2	Predicted R^2	Remarks
Linear	0.4698	0.0282	-0.0349	-0.6393	
2FI	0.5609	0.0248	-0.1230	-1.3947	
Quadratic	0.0177	0.0742	0.6868	-0.0754	Suggested
Cubic	0.9610	0.0261	0.4917	-8.2751	Aliased
Disintegration Time (Sec)					
Linear	0.6008	0.0001	-0.1005	-0.7754	
2FI	0.5008	0.0001	-0.1733	-1.4152	
Quadratic	0.0106	0.0005	0.9286	0.7715	Suggested
Cubic	0.8448	0.0002	0.6026	-6.6274	Aliased
CP : Avcl Formulations					
Friability (%)					
Source	*Sequential p-value*	Lack of Fit p-value	Adjusted R^2	Predicted R^2	Remarks
Linear	0.0002	0.7092	0.8453	0.7532	
2FI	0.9432	0.4077	0.8234	0.6305	
Quadratic	0.0300	0.3187	0.8892	0.5856	Suggested
Cubic	0.2331	0.4166	0.8616	0.2864	Aliased
Disintegration Time (Sec)					
Linear	0.0051	0.3316	0.6667	0.4226	
2FI	0.5942	0.3494	0.6353	0.3656	
Quadratic	0.0333	0.8861	0.8380	0.4240	Suggested
Cubic	0.1874	0.6330	0.8285	-0.3728	Aliased

Table S2: ANOVA results for second polynomial equations (CP: Avcl)

**Response 1: R1 (Friability)**						
**Source**	**Sum of Squares**	**df**	**Mean Square**	**F-value**	**p-value**	
**Model**	0.0123	5	0.0025	27.01	0.0013	significant
**A-CP**	0.0013	1	0.0013	14.73	0.0122	
**B-Avcl**	0.0023	1	0.0023	25.58	0.0039	
**AB**	0.0001	1	0.0001	1.10	0.3429	
**A^2**	0.0083	1	0.0083	90.57	0.0002	
**B^2**	0.0001	1	0.0001	1.24	0.3161	
**Residual**	0.0005	5	0.0001			

**Lack of Fit**	0.0004	3	0.0001	3.89	0.2112	not significant
**Pure Error**	0.0001	2	0.0000			
**Cor Total**	0.0127	10				
	**Fit Statistics**					
**Std. Dev.**	0.0095		**R^2**			0.9643
**Mean**	0.2723		**Adjusted R^2**			0.9286
**C.V. %**	3.51		**Predicted R^2**			0.7715
			**Adeq Precision**			15.5919
			**Response 2: R2 (Disintegration time)**			
**Source**	**Sum of Squares**	**df**	**Mean Square**	**F-value**	**p-value**	
**Model**	64.83	5	12.97	11.35	0.0093	significant
**A-POM**	39.09	1	39.09	34.20	0.0021	
**B-Avcl**	12.64	1	12.64	11.06	0.0209	
**AB**	0.4900	1	0.4900	0.4287	0.5415	
**A^2**	10.19	1	10.19	8.92	0.0306	
**B^2**	0.3045	1	0.3045	0.2664	0.6277	
**Residual**	0.5710	5	0.5710			
**Lack of Fit**	0.5710	3	0.5710	0.4995	0.4647	not significant
**Pure Error**	0.0000	2	0.0000			
**Cor Total**	64.34	10				
	**Fit Statistics**					
**Std. Dev.**	1.07		**R^2**			0.9190
**Mean**	15.19		**Adjusted R^2**			0.8380
**C.V. %**	7.04		**Predicted R^2**			0.4240
			**Adeq. Precision**			10.1983

EQUATIONS

$$R1 = 0.303+0.018X1+0.024X2+0.009X1X2-0.07X1^2-0.0089X2^2$$

$$R2 = 15.99-3.12X1+1.77X2+0.69X1X2-2.68X1^2+0.46X2^2$$
